# 3-[(*E*)-2,4-Dichloro­benzyl­idene]-1-methyl­piperidin-4-one

**DOI:** 10.1107/S1600536808002286

**Published:** 2008-01-25

**Authors:** D. Gayathri, D. Velmurugan, R. Ranjith Kumar, S. Perumal, K. Ravikumar

**Affiliations:** a Centre of Advanced Study in Crystallography and Biophysics, University of Madras, Guindy Campus, Chennai 600 025, India; b Department of Organic Chemistry, School of Chemistry, Madurai Kamaraj University, Madurai 625 021, India; c Laboratory of X-ray Crystallography, Indian Institute of Chemical Technology, Hyderabad 500 007, India

## Abstract

The piperidine ring of the title compound, C_13_H_13_Cl_2_NO, adopts an envelope conformation. Inter­molecular C—H⋯O inter­actions link the mol­ecules into a *C*(7) chain running along the *b* axis.

## Related literature

For biological activities of 4-piperidones, see: Badorrey *et al.* (1999 ▶); Grishina *et al.* (1994 ▶); Nalanishi *et al.* (1974*a* ▶,*b* ▶). For ring conformations, see: Cremer & Pople (1975 ▶); Nardelli (1983 ▶).
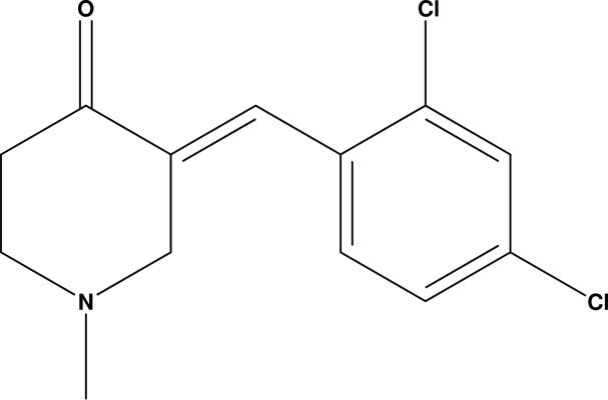


## Experimental

### 

#### Crystal data

C_13_H_13_Cl_2_NO
*M* *_r_* = 270.14
Monoclinic, 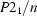

*a* = 12.2013 (9) Å
*b* = 8.5901 (6) Å
*c* = 12.6391 (9) Å
β = 92.997 (1)°
*V* = 1322.90 (16) Å^3^
*Z* = 4
Mo *K*α radiation
μ = 0.47 mm^−1^
*T* = 293 (2) K
0.24 × 0.23 × 0.20 mm

#### Data collection

Bruker SMART APEX CCD area-detector diffractometer
Absorption correction: none
14377 measured reflections
3071 independent reflections
2654 reflections with *I* > 2σ(*I*)
*R*_int_ = 0.018

#### Refinement

*R*[*F* ^2^ > 2σ(*F* ^2^)] = 0.040
*wR*(*F* ^2^) = 0.120
*S* = 0.97
3071 reflections
155 parameters
H-atom parameters constrained
Δρ_max_ = 0.34 e Å^−3^
Δρ_min_ = −0.28 e Å^−3^

### 

Data collection: *SMART* (Bruker, 2001 ▶); cell refinement: *SAINT* (Bruker, 2001 ▶); data reduction: *SAINT*; program(s) used to solve structure: *SHELXS97* (Sheldrick, 2008 ▶); program(s) used to refine structure: *SHELXL97* (Sheldrick, 2008 ▶); molecular graphics: *PLATON* (Spek, 2003 ▶); software used to prepare material for publication: *SHELXL97* and *PARST* (Nardelli, 1995 ▶).

## Supplementary Material

Crystal structure: contains datablocks I, global. DOI: 10.1107/S1600536808002286/ci2557sup1.cif

Structure factors: contains datablocks I. DOI: 10.1107/S1600536808002286/ci2557Isup2.hkl

Additional supplementary materials:  crystallographic information; 3D view; checkCIF report

## Figures and Tables

**Table 1:** Hydrogen-bond geometry (Å, °)

*D*—H⋯*A*	*D*—H	H⋯*A*	*D*⋯*A*	*D*—H⋯*A*
C13—H13⋯O1^i^	0.93	2.46	3.366 (2)	163

Symmetry code: (i) 
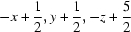
.

**Fig. 1.**
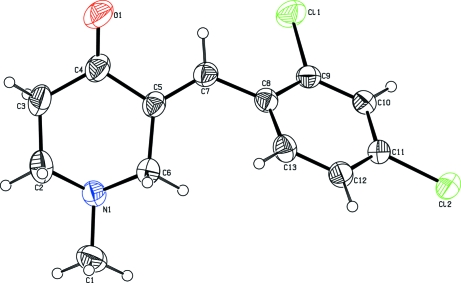
The molecular structure of the title compound, showing 30% probability displacement ellipsoids.

**Fig. 2.**
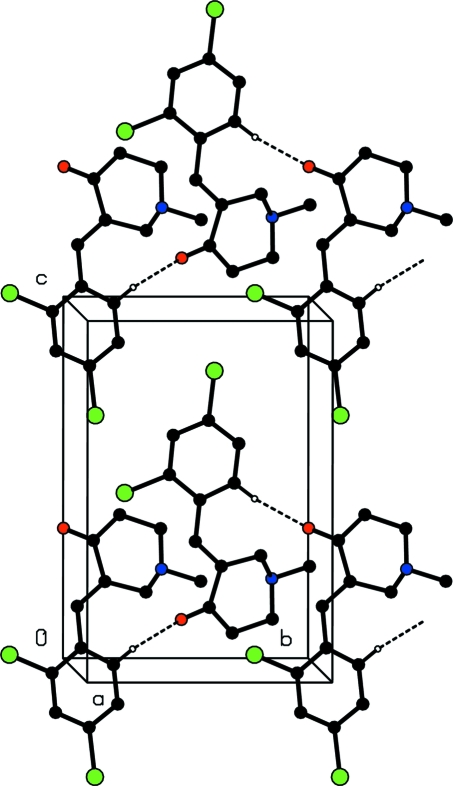
The molecular packing in the title compound, viewed approximately down the a axis.

## supplementary crystallographic information

### Comment

Synthesis of 4-piperidones is of current interest due to their potential medical applications (Grishina *et al.*, 1994). 4-Piperidones have been found to exhibit blood cholesterol-lowering activities (Nalanishi *et al.*, 1974*a*,*b*). Various piperidones and piperidine derivatives are present in numerous alkaloids (Badorrey *et al.*, 1999). As the title compound is of biological significance, the crystal structure of the title compound has been determined by X-ray diffraction.

The sum of the bond angles around atom N1 (330 °) indicates *sp*^3^– hybridization. Atoms Cl1 and Cl2 deviate from the plane of the attached benzene ring by 0.075 (1) and -0.094 (1) Å, respectively. The piperidine ring adopts an envelope conformation, with puckering parameters (Cremer & Pople, 1975) and smallest displacement asymmetry parameters (Nardelli, 1983) of Q = 0.504 (2) Å, θ = 141.1 (2)°, φ = 193.7 (4)° and ΔC_s_[N1] = 8.7 (2)°.

In the crystal structure, the C—H···O intermolecular interactions generate a C(7) chain running along the *b* axis.

### Experimental

A mixture of 1-methyl-4-piperidone (1 mmol) and pyrrolidine (1.2 mmol) was taken in a glass tube, mixed well and kept aside for 5 min at ambient temperature. To this mixture, 2,4-dichlorobenzaldehyde (1 mmol) was added, mixed thoroughly and the tube containing the mixture was partially immersed in a silica bath placed in a microwave oven and irradiated at 4 power level for 7 minutes. The progress of the reaction was monitored after every 1 min of irradiation by TLC with petroleum ether:ethyl acetate (1:2 *v*/*v* mixture) as eluent. fter each irradiation, the reaction mixture was cooled to room temperature and mixed well. The maximum temperature of the silica bath, measured immediately after each irradiation was over by stirring the silica bath with the thermometer, was found to be 338 K. After completion of the reaction as evident from the TLC, the product was purified by column chromatography using petroleum ether:ethyl acetate (7:2 *v*/*v*) mixture and crystallized from ethyl acetate.

### Refinement

H atoms were positioned geometrically and allowed to ride on their parent atoms, with C—H = 0.93–0.97 Å and *U*_iso_(H) = 1.5*U*_eq_(methyl C) or 1.2*U*_eq_(C). A rotating group model was used for the methyl groups.

### Crystal data

**Table:**

C_13_H_13_Cl_2_NO	*F*_000_ = 560
*M**_r_* = 270.14	*D*_x_ = 1.356 Mg m^−^^3^
Monoclinic, *P*2_1_/*n*	Mo *K*α radiation λ = 0.71073 Å
Hall symbol: -P 2yn	Cell parameters from 1509 reflections
*a* = 12.2013 (9) Å	θ = 2.3–25.0º
*b* = 8.5901 (6) Å	µ = 0.47 mm^−^^1^
*c* = 12.6391 (9) Å	*T* = 293 (2) K
β = 92.997 (1)º	Block, pale yellow
*V* = 1322.90 (16) Å^3^	0.24 × 0.23 × 0.20 mm
*Z* = 4	

### Data collection

**Table:**

Bruker SMART APEX CCD area-detector diffractometer	2654 reflections with *I* > 2σ(*I*)
Radiation source: fine-focus sealed tube	*R*_int_ = 0.019
Monochromator: graphite	θ_max_ = 28.1º
*T* = 293(2) K	θ_min_ = 2.3º
ω scans	*h* = −15→15
Absorption correction: none	*k* = −10→11
14377 measured reflections	*l* = −16→16
3071 independent reflections	

### Refinement

**Table:**

Refinement on *F*^2^	Secondary atom site location: difference Fourier map
Least-squares matrix: full	Hydrogen site location: inferred from neighbouring sites
*R*[*F*^2^ > 2σ(*F*^2^)] = 0.040	H-atom parameters constrained
*wR*(*F*^2^) = 0.120	*w* = 1/[σ^2^(*F*_o_^2^) + (0.0724*P*)^2^ + 0.3356*P*] where *P* = (*F*_o_^2^ + 2*F*_c_^2^)/3
*S* = 0.97	(Δ/σ)_max_ = 0.001
3071 reflections	Δρ_max_ = 0.34 e Å^−^^3^
155 parameters	Δρ_min_ = −0.27 e Å^−^^3^
Primary atom site location: structure-invariant direct methods	Extinction correction: none

### Special details

**Table:**

Geometry. All e.s.d.'s (except the e.s.d. in the dihedral angle between two l.s. planes) are estimated using the full covariance matrix. The cell e.s.d.'s are taken into account individually in the estimation of e.s.d.'s in distances, angles and torsion angles; correlations between e.s.d.'s in cell parameters are only used when they are defined by crystal symmetry. An approximate (isotropic) treatment of cell e.s.d.'s is used for estimating e.s.d.'s involving l.s. planes.
Refinement. Refinement of *F*^2^ against ALL reflections. The weighted *R*-factor *wR* and goodness of fit *S* are based on *F*^2^, conventional *R*-factors *R* are based on *F*, with *F* set to zero for negative *F*^2^. The threshold expression of *F*^2^ > σ(*F*^2^) is used only for calculating *R*-factors(gt) *etc*. and is not relevant to the choice of reflections for refinement. *R*-factors based on *F*^2^ are statistically about twice as large as those based on *F*, and *R*- factors based on ALL data will be even larger.

### Fractional atomic coordinates and isotropic or equivalent isotropic displacement parameters (Å^2^)

**Table:**

	*x*	*y*	*z*	*U*_iso_*/*U*_eq_	
C1	0.53390 (18)	0.5088 (2)	1.24853 (18)	0.0781 (5)	
H1A	0.5518	0.5079	1.1755	0.117*	
H1B	0.5966	0.5421	1.2917	0.117*	
H1C	0.4740	0.5792	1.2576	0.117*	
C2	0.47091 (17)	0.3512 (2)	1.38999 (14)	0.0700 (5)	
H2A	0.4038	0.4104	1.3959	0.084*	
H2B	0.5280	0.4004	1.4346	0.084*	
C3	0.4536 (2)	0.1860 (3)	1.42729 (13)	0.0791 (6)	
H3A	0.5248	0.1374	1.4403	0.095*	
H3B	0.4182	0.1896	1.4942	0.095*	
C4	0.38604 (15)	0.0855 (2)	1.35183 (13)	0.0660 (5)	
C5	0.37681 (12)	0.1336 (2)	1.23733 (11)	0.0524 (3)	
C6	0.41053 (13)	0.29745 (19)	1.21048 (12)	0.0532 (3)	
H6A	0.4317	0.3008	1.1376	0.064*	
H6B	0.3484	0.3667	1.2168	0.064*	
C7	0.34326 (12)	0.0240 (2)	1.16663 (12)	0.0545 (4)	
H7	0.3257	−0.0728	1.1939	0.065*	
C8	0.33134 (11)	0.04126 (18)	1.05058 (11)	0.0486 (3)	
C9	0.35991 (12)	−0.08015 (17)	0.98306 (12)	0.0489 (3)	
C10	0.35760 (12)	−0.06504 (17)	0.87383 (12)	0.0496 (3)	
H10	0.3791	−0.1465	0.8311	0.059*	
C11	0.32225 (13)	0.07535 (18)	0.83036 (11)	0.0500 (3)	
C12	0.28628 (14)	0.19534 (19)	0.89226 (13)	0.0565 (4)	
H12	0.2592	0.2867	0.8613	0.068*	
C13	0.29116 (13)	0.17732 (19)	1.00152 (13)	0.0557 (4)	
H13	0.2670	0.2581	1.0434	0.067*	
N1	0.50222 (11)	0.35202 (16)	1.28025 (10)	0.0539 (3)	
O1	0.34393 (15)	−0.0325 (2)	1.38258 (12)	0.1014 (6)	
Cl1	0.40318 (5)	−0.25836 (5)	1.03652 (4)	0.07721 (18)	
Cl2	0.32208 (5)	0.09909 (5)	0.69335 (3)	0.07158 (17)	

### Atomic displacement parameters (Å^2^)

**Table:**

	*U*^11^	*U*^22^	*U*^33^	*U*^12^	*U*^13^	*U*^23^
C1	0.0843 (13)	0.0546 (10)	0.0934 (14)	−0.0057 (9)	−0.0158 (11)	0.0027 (10)
C2	0.0752 (11)	0.0799 (12)	0.0544 (9)	0.0079 (9)	−0.0035 (8)	−0.0182 (9)
C3	0.0949 (14)	0.1009 (15)	0.0412 (8)	−0.0155 (12)	0.0012 (8)	0.0010 (9)
C4	0.0617 (9)	0.0896 (13)	0.0472 (8)	−0.0133 (9)	0.0065 (7)	0.0089 (8)
C5	0.0465 (7)	0.0672 (9)	0.0437 (7)	−0.0049 (6)	0.0037 (6)	0.0033 (6)
C6	0.0523 (8)	0.0595 (9)	0.0475 (7)	0.0023 (7)	−0.0001 (6)	−0.0007 (7)
C7	0.0509 (8)	0.0625 (9)	0.0501 (8)	−0.0084 (7)	0.0038 (6)	0.0046 (7)
C8	0.0424 (7)	0.0541 (8)	0.0493 (7)	−0.0043 (6)	0.0011 (5)	−0.0007 (6)
C9	0.0473 (7)	0.0448 (7)	0.0543 (8)	−0.0020 (6)	−0.0011 (6)	0.0045 (6)
C10	0.0512 (7)	0.0460 (7)	0.0513 (8)	−0.0012 (6)	0.0016 (6)	−0.0035 (6)
C11	0.0535 (8)	0.0504 (7)	0.0455 (7)	−0.0036 (6)	−0.0018 (6)	0.0003 (6)
C12	0.0639 (9)	0.0483 (8)	0.0563 (8)	0.0069 (7)	−0.0084 (7)	−0.0006 (7)
C13	0.0561 (8)	0.0555 (8)	0.0549 (8)	0.0071 (7)	−0.0032 (6)	−0.0090 (7)
N1	0.0546 (7)	0.0524 (7)	0.0540 (7)	0.0017 (5)	−0.0029 (5)	−0.0036 (5)
O1	0.1116 (12)	0.1262 (13)	0.0654 (8)	−0.0543 (11)	−0.0048 (8)	0.0325 (9)
Cl1	0.1059 (4)	0.0545 (3)	0.0708 (3)	0.0144 (2)	0.0005 (3)	0.01340 (19)
Cl2	0.1041 (4)	0.0622 (3)	0.0482 (2)	−0.0037 (2)	0.0024 (2)	0.00406 (17)

### Geometric parameters (Å, °)

**Table:**

C1—N1	1.463 (2)	C6—H6A	0.97
C1—H1A	0.96	C6—H6B	0.97
C1—H1B	0.96	C7—C8	1.474 (2)
C1—H1C	0.96	C7—H7	0.93
C2—N1	1.458 (2)	C8—C13	1.400 (2)
C2—C3	1.514 (3)	C8—C9	1.403 (2)
C2—H2A	0.97	C9—C10	1.385 (2)
C2—H2B	0.97	C9—Cl1	1.7440 (15)
C3—C4	1.501 (3)	C10—C11	1.385 (2)
C3—H3A	0.97	C10—H10	0.93
C3—H3B	0.97	C11—C12	1.380 (2)
C4—O1	1.209 (2)	C11—Cl2	1.7435 (15)
C4—C5	1.504 (2)	C12—C13	1.388 (2)
C5—C7	1.346 (2)	C12—H12	0.93
C5—C6	1.510 (2)	C13—H13	0.93
C6—N1	1.465 (2)		
			
N1—C1—H1A	109.5	N1—C6—H6B	109.3
N1—C1—H1B	109.5	C5—C6—H6B	109.3
H1A—C1—H1B	109.5	H6A—C6—H6B	107.9
N1—C1—H1C	109.5	C5—C7—C8	126.95 (15)
H1A—C1—H1C	109.5	C5—C7—H7	116.5
H1B—C1—H1C	109.5	C8—C7—H7	116.5
N1—C2—C3	110.39 (15)	C13—C8—C9	116.34 (13)
N1—C2—H2A	109.6	C13—C8—C7	122.59 (14)
C3—C2—H2A	109.6	C9—C8—C7	121.07 (14)
N1—C2—H2B	109.6	C10—C9—C8	122.94 (13)
C3—C2—H2B	109.6	C10—C9—Cl1	117.27 (11)
H2A—C2—H2B	108.1	C8—C9—Cl1	119.78 (12)
C4—C3—C2	114.99 (16)	C11—C10—C9	117.82 (13)
C4—C3—H3A	108.5	C11—C10—H10	121.1
C2—C3—H3A	108.5	C9—C10—H10	121.1
C4—C3—H3B	108.5	C12—C11—C10	121.83 (14)
C2—C3—H3B	108.5	C12—C11—Cl2	119.47 (12)
H3A—C3—H3B	107.5	C10—C11—Cl2	118.69 (12)
O1—C4—C5	121.85 (17)	C11—C12—C13	118.85 (15)
O1—C4—C3	120.41 (16)	C11—C12—H12	120.6
C5—C4—C3	117.69 (16)	C13—C12—H12	120.6
C7—C5—C4	116.86 (16)	C12—C13—C8	121.99 (14)
C7—C5—C6	125.39 (14)	C12—C13—H13	119.0
C4—C5—C6	117.70 (14)	C8—C13—H13	119.0
N1—C6—C5	111.81 (13)	C1—N1—C2	110.56 (15)
N1—C6—H6A	109.3	C1—N1—C6	109.51 (14)
C5—C6—H6A	109.3	C2—N1—C6	109.96 (13)
			
N1—C2—C3—C4	−46.3 (2)	C13—C8—C9—Cl1	−176.27 (11)
C2—C3—C4—O1	−161.3 (2)	C7—C8—C9—Cl1	3.54 (19)
C2—C3—C4—C5	21.1 (3)	C8—C9—C10—C11	−2.0 (2)
O1—C4—C5—C7	−15.5 (3)	Cl1—C9—C10—C11	179.32 (11)
C3—C4—C5—C7	162.09 (18)	C9—C10—C11—C12	−2.4 (2)
O1—C4—C5—C6	167.14 (19)	C9—C10—C11—Cl2	178.30 (11)
C3—C4—C5—C6	−15.2 (2)	C10—C11—C12—C13	3.4 (2)
C7—C5—C6—N1	−142.95 (16)	Cl2—C11—C12—C13	−177.33 (13)
C4—C5—C6—N1	34.12 (19)	C11—C12—C13—C8	0.0 (2)
C4—C5—C7—C8	−177.87 (14)	C9—C8—C13—C12	−4.0 (2)
C6—C5—C7—C8	−0.8 (3)	C7—C8—C13—C12	176.22 (14)
C5—C7—C8—C13	−38.6 (2)	C3—C2—N1—C1	−172.10 (17)
C5—C7—C8—C9	141.58 (17)	C3—C2—N1—C6	66.9 (2)
C13—C8—C9—C10	5.0 (2)	C5—C6—N1—C1	178.07 (14)
C7—C8—C9—C10	−175.16 (13)	C5—C6—N1—C2	−60.26 (17)

### Hydrogen-bond geometry (Å, °)

**Table:**

*D*—H···*A*	*D*—H	H···*A*	*D*···*A*	*D*—H···*A*
C13—H13···O1^i^	0.93	2.46	3.366 (2)	163

Symmetry codes: (i) −*x*+1/2, *y*+1/2, −*z*+5/2.
